# Expiratory model-based method to monitor ARDS disease state

**DOI:** 10.1186/1475-925X-12-57

**Published:** 2013-06-26

**Authors:** Erwin J van Drunen, Yeong Shiong Chiew, J Geoffrey Chase, Geoffrey M Shaw, Bernard Lambermont, Nathalie Janssen, Nor Salwa Damanhuri, Thomas Desaive

**Affiliations:** 1 University of Canterbury, 8041, Christchurch, New Zealand; 2 Christchurch Hospital, 8011, Christchurch, New Zealand; 3 University Hospital of Liège, Liège, Belgium; 4 University of Liège, Liège, Belgium

**Keywords:** Mechanical ventilation, Model-based methods, Expiration, ARDS, Intensive care, Time constant

## Abstract

**Introduction:**

Model-based methods can be used to characterise patient-specific condition and response to mechanical ventilation (MV) during treatment for acute respiratory distress syndrome (ARDS). Conventional metrics of respiratory mechanics are based on inspiration only, neglecting data from the expiration cycle. However, it is hypothesised that expiratory data can be used to determine an alternative metric, offering another means to track patient condition and guide positive end expiratory pressure (PEEP) selection.

**Methods:**

Three fully sedated, oleic acid induced ARDS piglets underwent three experimental phases. Phase 1 was a healthy state recruitment manoeuvre. Phase 2 was a progression from a healthy state to an oleic acid induced ARDS state. Phase 3 was an ARDS state recruitment manoeuvre. The expiratory time-constant model parameter was determined for every breathing cycle for each subject. Trends were compared to estimates of lung elastance determined by means of an end-inspiratory pause method and an integral-based method. All experimental procedures, protocols and the use of data in this study were reviewed and approved by the Ethics Committee of the University of Liege Medical Faculty.

**Results:**

The overall median absolute percentage fitting error for the expiratory time-constant model across all three phases was less than 10 %; for each subject, indicating the capability of the model to capture the mechanics of breathing during expiration. Provided the respiratory resistance was constant, the model was able to adequately identify trends and fundamental changes in respiratory mechanics.

**Conclusion:**

Overall, this is a proof of concept study that shows the potential of continuous monitoring of respiratory mechanics in clinical practice. Respiratory system mechanics vary with disease state development and in response to MV settings. Therefore, titrating PEEP to minimal elastance theoretically results in optimal PEEP selection. Trends matched clinical expectation demonstrating robustness and potential for guiding MV therapy. However, further research is required to confirm the use of such real-time methods in actual ARDS patients, both sedated and spontaneously breathing.

## Background

Patients suffering from severe respiratory insufficiency, such as acute respiratory distress syndrome (ARDS) [1] are admitted to the intensive care unit (ICU) and require mechanical ventilation (MV) for breathing support. ARDS is associated with a loss of functional lung units resulting in a stiffer lung [2]. The severity of ARDS is typically measured as the ratio of the arterial partial pressure of oxygen divided by the fraction of inspired oxygen (PaO_2_/FiO_2_) [1]. Clinicians offer a supportive environment to ARDS patients by applying positive end expiratory pressure (PEEP) to aid recovery by improving gas exchange and maintaining recruitment during subsequent breathing cycles [3-6]. Given the impact of MV on cost and length of stay [3], ensuring optimal PEEP would have significant impact.

Modelling the respiratory mechanics of MV patients can potentially provide a non-invasive method to obtain clinically and physiologically useful information to guide MV therapy [7-10]. Conventional metrics of respiratory mechanics are estimated based on the mechanics of breathing during inspiration, often neglecting expiratory data. However, passive expiration can be used to determine a metric based on the expiratory flow profile [11], potentially offering insight into respiratory mechanics in situations where conventional inspiratory metrics will not work. Real-time monitoring of model-based respiratory mechanics throughout treatment for ARDS will provide unique descriptions of the patient’s disease progression and response to MV [12-15], offering the ability to guide patient-specific MV.

## Method

### Time-constant model

The single compartment lung model is defined [16]:

(1) $$P_{\mathrm{aw}}\left(t\right)=R_{\mathrm{rs}}\times Q\left(t\right)+E_{\mathrm{rs}}\times V\left(t\right)+P_{0}$$

where *P*_*aw*_ is the airway pressure, *t* is time, *R*_*rs*_ is the series resistance of the endotracheal tube and the conducting airway, *Q* is the air flow, *E*_*rs*_ is the respiratory system elastance, *V* is the lung volume and *P*_*0*_ is the offset pressure.

Inspiration and expiration are different physiological processes. Expiration is essentially the passive unloading of the inspired tidal volume over a resistance at a constant ventilator applied pressure (*P*_*aw*_ = PEEP) with *P*_*0*_ ≈ PEEP [17,18]. Noting that volume is the integral of flow with respect to time, Eq. 1 in expiration becomes:

(2) $$\mathrm{PEEP}=R_{\mathrm{rs}}\times Q\left(t\right)+E_{\mathrm{rs}}\times \int_{0}^{t}Q\;\left(t^{'}\right)dt^{'}+\mathrm{PEEP}$$

Differentiating Eq. 2 yields:

(3) $$0=R_{\mathrm{rs}}\times \frac{\mathrm{dQ}\left(t\right)}{\mathrm{dt}}+E_{\mathrm{rs}}\times Q\left(t\right)$$

Dividing Eq. 3 by the resistance, *R*_*rs*_, yields a simple ordinary differential equation:

(4) $$\frac{\mathrm{dQ}\left(t\right)}{\mathrm{dt}}+\frac{E_{\mathrm{rs}}}{R_{\mathrm{rs}}}\times Q\left(t\right)=0$$

Solving Eq. 4 yields:

(5) Qt=Qoe−tτ=Qoe−Kt

where *Q*_*o*_ is the value of maximum expiratory flow and *τ* = 1/*K* = *R*_*rs*_/*E*_*rs*_ is the system time-constant [11].

**Figure 1:**
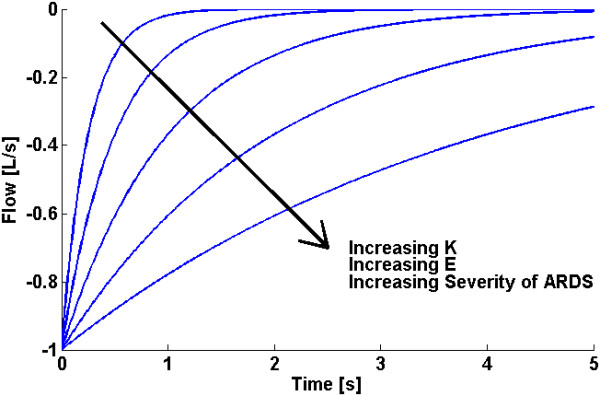
**Example of how changes in expiratory flow profile over time may be used to determine changes in a patients’ disease state, assuming *R***_***rs***_**is constant.**

If the resistance, *R*_*rs*_, is assumed constant [19], then *K* is directly proportional to *E*_*rs*_ where an increasing parameter *K* implies a less compliant lung as ARDS progresses, as shown in Figure 1.

### Experimental data

This study examines three fully sedated experimental ARDS piglets ventilated using Engström CareStation ventilators (Datex, General Electric Healthcare, Finland) with a volume controlled, square flow profile and a constant FiO_2_ of 0.5 [20]. An Eview data acquisition device with a constant sampling rate was used to record the airway pressure and flow data continuously throughout the trials.

The subjects underwent three experimental phases. Phase 1 was a staircase recruitment manoeuvre with PEEP settings at 5 – 10 – 15 – 20 – 15 – 10 – 5 cmH_2_O while the subject was in a healthy state. Phase 2 was a progression from a healthy sedated state to an oleic acid induced ARDS state at a constant PEEP of 5 cmH_2_O. Phase 3 was a staircase recruitment manoeuvre with PEEP settings at 5 – 10 – 15 – 20 – 15 – 10 – 5 cmH_2_O after the subject was diagnosed with ARDS. Thus, an initial recruitment manoeuvre provides a comparison to a healthy state. Each subject has between 1600 and 3500 recorded breathing cycles across all three phases for a total of 6800 breathing cycles. All experimental procedures, protocols and the use of data in this study were reviewed and approved by the Ethics Committee of the University of Liege Medical Faculty.

### Model fitting

**Figure 2:**
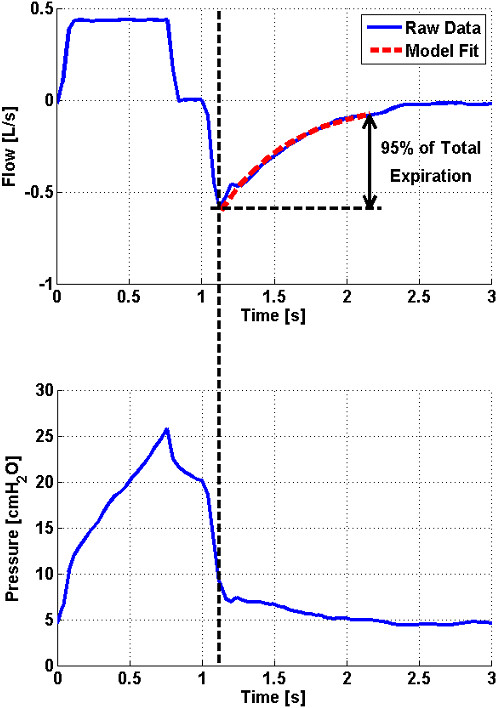
**Top: Flow profile of a single breathing cycle with exponential model fit to expiratory flow data.** Bottom: Pressure profile of a single breathing cycle at PEEP = 5 cmH_2_O. In this case, pressure data after 1.12 seconds is physiologically meaningless.

The values of *K* and *Q*_*o*_ are determined from the least-squares best fit of the time-constant model (Eq. 5) to the expiratory flow data. Determining *Q*_*o*_ simultaneously with *K* leads to robust parameter identification as the effect of outliers at the beginning of expiration is reduced. During expiration, the density of data points increases as expiration progresses because the rate of change of flow decreases. Therefore, the model fit becomes more constrained towards the end of expiration. When combined with the presence of a long portion of near-constant flow at the end of expiration caused by resistance in the ventilator’s expiratory valve [11], poor model fitting can occur. Hence, model fitting is limited to the time span required for the respiratory system to reach 95%; of its equilibrium value, as illustrated in Figure 2 (Top).

The expiratory pressure data recorded in this study shows a sudden decrease to just above PEEP, followed by a small trailing portion as shown for a representative breath in Figure 2 (Bottom). This data is expected to contain no physiologically useful information since it is measured downstream of the ventilator’s expiratory valve. Furthermore, the change in pressure from 1.12 seconds to the end of expiration is minor. This outcome further justifies the approach of only considering flow data for model fitting during expiration.

### Validation

The time-constant model parameter, *K*, is determined continuously for every breathing cycle for each subject at different experimental phases. The model is validated by comparing trends in *K* to trends obtained using an end-inspiratory pause (EIP) method and trends obtained using an integral-based method. Both methods determine unique inspiratory values of respiratory elastance and resistance for each breathing cycle, providing more insight into respiratory mechanics than the single lumped parameter, *K*. In particular:

1 The EIP method determines metrics of respiratory mechanics directly from the Engström CareStation ventilator (Datex, General Electric Healthcare, Finland) which automates a short EIP during controlled MV [21-23]. The zero-flow phase during EIP omits the resistance component in Eq. 1 and prolongs inspiration, allowing the inspired tidal volume (*V*_*t*_) to distribute evenly in the lung. The resulting pressure after the EIP is called the plateau pressure, *P*_*plat*_, and can be used to estimate static ventilation elastance, *E*_*static*_, as shown in Eq. 6. Equally, the pressure difference between peak inspiratory pressure (PIP) and *P*_*plat*_ can be used to calculate static airway resistance, *R*_*static*_, as shown in Eq. 7.

(6) $$E_{\mathrm{static}}=\left(P_{\mathrm{plat}}–\mathrm{PEEP}\right)/V_{t}$$

(7) $$R_{\mathrm{static}}=\left(\mathrm{PIP}–P_{\mathrm{plat}}\right)/Q$$

2 Eq. 8 describes an integral-based method [24] to estimate breath-specific values of the respiratory elastance, *E*_*rs*_, and respiratory resistance, *R*_*rs*_. The recorded inspiratory pressure and flow data for each breath is used to determine values that best fit the single compartment lung model defined in Eq. 1. Integral-based parameter identification is similar to multiple linear regression where using integrals significantly increases robustness to noise [19,24].

(8) $$\int P_{\mathrm{aw}}\left(t\right)=R_{\mathrm{rsIB}}\times \int Q\left(t\right)+E_{\mathrm{rsIB}}\times \int V\left(t\right)+\int P_{0}$$

**Figure 3:**
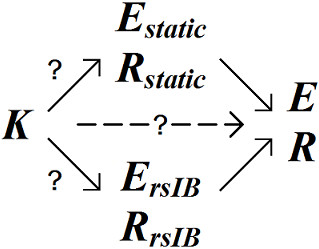
Graphic representation of the relationship between various metrics of respiratory mechanics.

Thus, *K* can be compared with, *E*_*static*_ and *R*_*static*_, as well as, *E*_*rsIB*_ and *R*_*rsIB*_, both of which aim to capture the true elastance (*E*) and resistance (*R*) of the lung. This validation process is summarised graphically in Figure 3.

## Results and discussion

### Fitting error

For each breath, the time-constant model and both validation methods are compared to recorded flow and pressure data respectively. In particular, the time-constant model parameters, *K* and *Q*_*o*_, are substituted into Eq. 5 and the calculated flow is compared to the measured expiratory flow data used during model fitting. In the case of both validation methods, the estimated parameters, *E*_*static*_ and *R*_*static*_, and, *E*_*rsIB*_ and *R*_*rsIB*_, are substituted into Eq. 1 and the calculated pressure is compared to the measured inspiratory pressure data. Median and inter-quartile range (IQR) absolute percentage fitting errors are reported in Table 1. The integral-based method produced the lowest overall median fitting error for each subject (1.97 %;, 1.55 %; and 2.26 %; for Subjects 1, 2 and 3 respectively). The time-constant model produced the second lowest overall median fitting error for Subjects 2 and 3. All overall median fitting errors were within likely measurement errors of 3-10 %;.

**Table 1:** Model fitting errors for each model/method

		**Absolute percentage fitting error (Median, [IQR]) [%;]**			
	**Subject**	**Phase 1**	**Phase 2**	**Phase 3**	**Overall**
**Time-Constant Model**	**1**	5.01	7.24	5.19	6.99
		[2.24-9.89]	[3.74-12.58]	[2.50-8.96]	[3.55-12.21]
	**2**	3.69	3.66	5.84	3.83
		[1.68-7.58]	[1.59-6.78]	[2.77-9.62]	[1.67-7.12]
	**3**	4.38	4.37	10.57	4.72
		[1.90-8.71]	[2.00-8.41]	[5.11-17.29]	[2.13-9.34]
**EIP Method**	**1**	2.61	3.85	4.07	3.77
		[1.25-4.87]	[1.85-6.99]	[1.75-7.83]	[1.80-6.92]
	**2**	5.83	5.31	6.81	5.48
		[3.28-10.26]	[2.69-9.13]	[3.84-9.91]	[2.83-9.34]
	**3**	5.59	6.32	12.71	6.61
		[3.58-9.91]	[3.51-11.04]	[6.51-24.32]	[3.65-11.69]
**Integral-Based Method**	**1**	1.42	1.98	2.31	1.97
		[0.59-3.18]	[0.92-3.84]	[1.03-4.12]	[0.90-3.81]
	**2**	1.81	1.48	2.04	1.55
		[0.85-3.27]	[0.71-2.86]	[0.93-3.43]	[0.73-2.97]
	**3**	1.31	2.23	4.99	2.26
		[0.68-2.36]	[0.95-4.95]	[2.14-8.59]	[0.96-5.11]

### Healthy State Recruitment Manoeuvre – Phase 1

**Figure 4:**
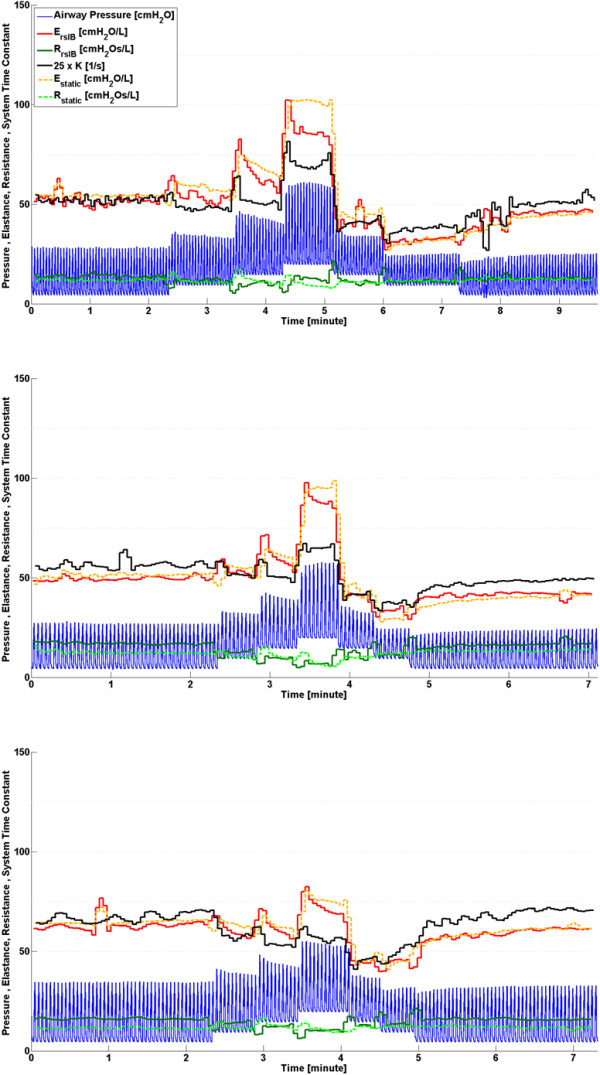
**Respiratory system mechanics monitoring during phase 1, healthy state recruitment manoeuvre.** Note that values of *K* have been scaled for clarity and serve only as an indication for trend comparison. Subject 1 (Top), Subject 2 (Middle) and Subject 3 (Bottom).

During the recruitment manoeuvre, *K* closely follows the trends of both *E*_*rsIB*_ and *E*_*static*_ for all three subjects, as shown in Figure 4. Estimates of resistance remain relatively constant across the recruitment manoeuvre justifying the use of the lumped parameter *K* during this phase.

### Disease Progression - Phase 2

**Figure 5:**
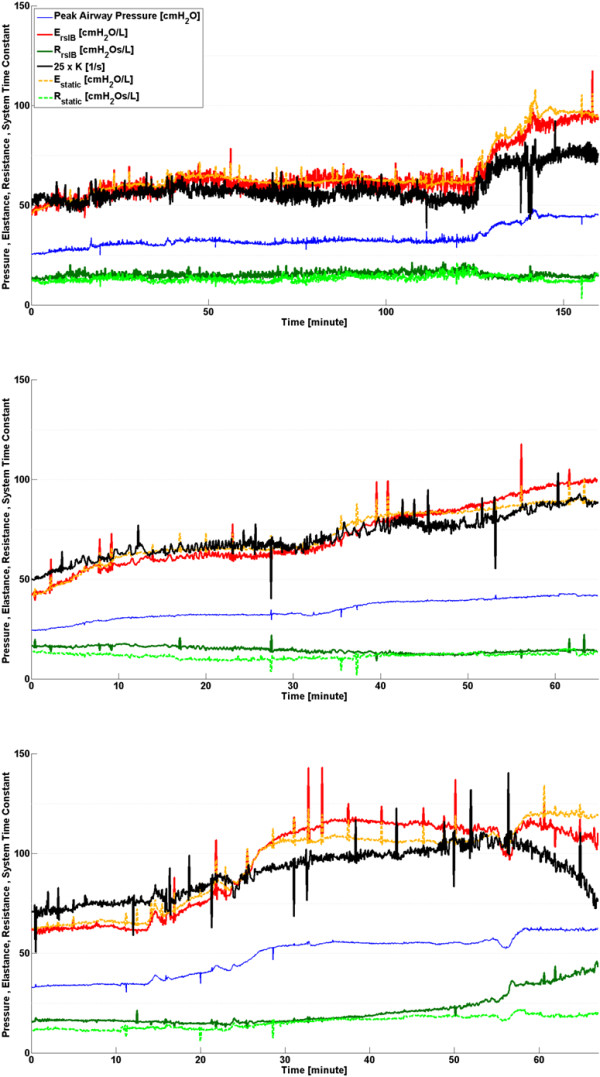
**Respiratory system mechanics monitoring during phase 2, disease progression.** Note that values of *K* have been scaled for clarity and serve only as an indication for trend comparison. Subject 1 (Top), Subject 2 (Middle) and Subject 3 (Bottom).

Inter-subject differences in ARDS progression over time can be seen in Figure 5 and indicate a variable response to oleic acid to induce ARDS, as well as a variable response to MV for each subject [25-28]. After oleic acid injection, it was found that *E*_*rsIB*_, *E*_*static*_ and *K* in Subjects 1 and 2 all followed similar trends. Each parameter shows a slow change, followed by a rapid change as ARDS develops, where increasing elastance implies the lung is becoming stiffer [2,29]. However, in Subject 3, the trend of *K* does not follow either *E*_*rsIB*_, *E*_*static*_. This lack of correlation may be a subject-specific response due to the increasing severity of ARDS closing some of the respiratory system airways, thereby increasing the resistance [2]. Since *K* = *E*_*rs*_/*R*_*rs*_, an increasing resistance would result in a decreasing *K*, consistent with that shown in Figure 5. In addition, because the resistance is varying for Subject 3, no information about the lung elasticity can be directly gained from *K* during this phase. This result highlights the importance of the assumption of constant resistance for accurate tracking of disease progression when using this method.

### ARDS state recruitment manoeuvre - Phase 3

**Figure 6:**
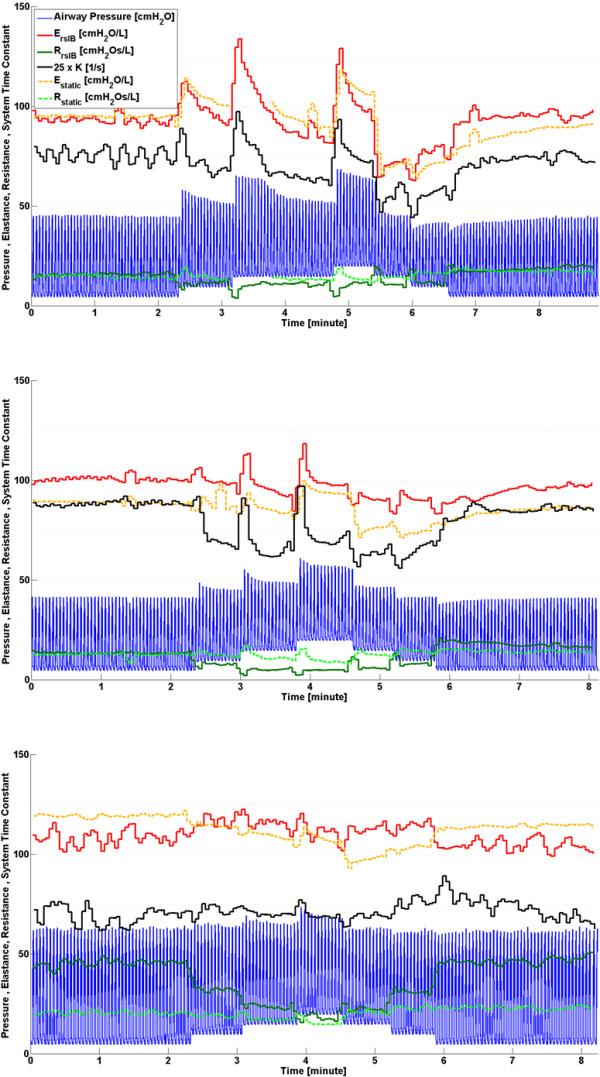
**Respiratory system mechanics monitoring during phase 3, disease state recruitment manoeuvre.** Note that values of *K* have been scaled for clarity and serve only as an indication for trend comparison. Subject 1 (Top), Subject 2 (Middle) and Subject 3 (Bottom).

Subject responses during phase 3 vary significantly as shown in Figure 6. Subject 1 has the highest variation in *E*_*rsIB*_, *E*_*static*_ and *K*, while Subject 3 does not show any significant change in either *E*_*rsIB*_ or *K*. Trends of *E*_*rsIB*_, *E*_*static*_ and *K* for both Subjects 1 and 2 agree, and show a definite response to the recruitment manoeuvre. Subject 3, and to a lesser extent Subject 2, show a decrease in *R*_*rsIB*_ during increasing PEEP titration and an increase in *R*_*rsIB*_ during decreasing PEEP titration, suggesting that increasing PEEP opens the respiratory system airways [12,14,30]. This change of airway resistance indirectly affects the estimated parameters determined by the integral-based method. Furthermore, Subject 3 has the highest PIP across all three phases. This is because a higher inspiratory pressure is required to counter the effect of a larger body mass (see Table 2). However, Subject 3 also has the highest severity of ARDS as shown in Table 2. Thus, it is possible that factors aside from a variable respiratory resistance may influence the response. This result highlights both the significant inter-subject variability, and the need for subject-specificity in a model-based approach.

**Table 2:** **Body mass and PaO**_**2**_**/FiO**_**2 **_**ratio (during ARDS state recruitment manoeuvre) for each subject**

**Subject**	**Mass [kg]**	**PaO**_**2**_**/FiO**_**2 **_**(Phase 3) [mmHg]**
**1**	24.0	126.6
**2**	20.3	183.6
**3**	29.6	113.6

During PEEP titration in phase 3, respiratory elastance drops to an overall minimum at a specific PEEP for each subject (PEEP = 15 cmH_2_O for Subject 1, PEEP = 10-15 cmH_2_O for Subject 2 and PEEP = 15-20 cmH_2_O for Subject 3). Because recruitment is a function of PEEP and time [31,32], true minimal *E*_*rsIB*_, *E*_*static*_ and *K* can only be determined after a stabilisation period at each PEEP level. Decrease of elastance over time to a specific minimum can be described by increasing recruitment and/or the lung’s viscoelastic properties, which causes hysteresis [33,34]. Setting PEEP at minimum elastance theoretically benefits ventilation by maximising recruitment, reducing work of breathing and avoiding overdistension [12,14,15,35]. It was also found that decreasing PEEP titration resulted in lower overall *E*_*rsIB*_, *E*_*static*_ and *K* compared to increasing PEEP titration, as shown in Figure 6. When PEEP is increased to a higher level, recruitment, as well as potential lung overstretching, occurs. However, after PEEP is reduced, the lung remains more compliant, as expected clinically after such a recruitment manoeuvre.

### Trend comparison

**Figure 7:**
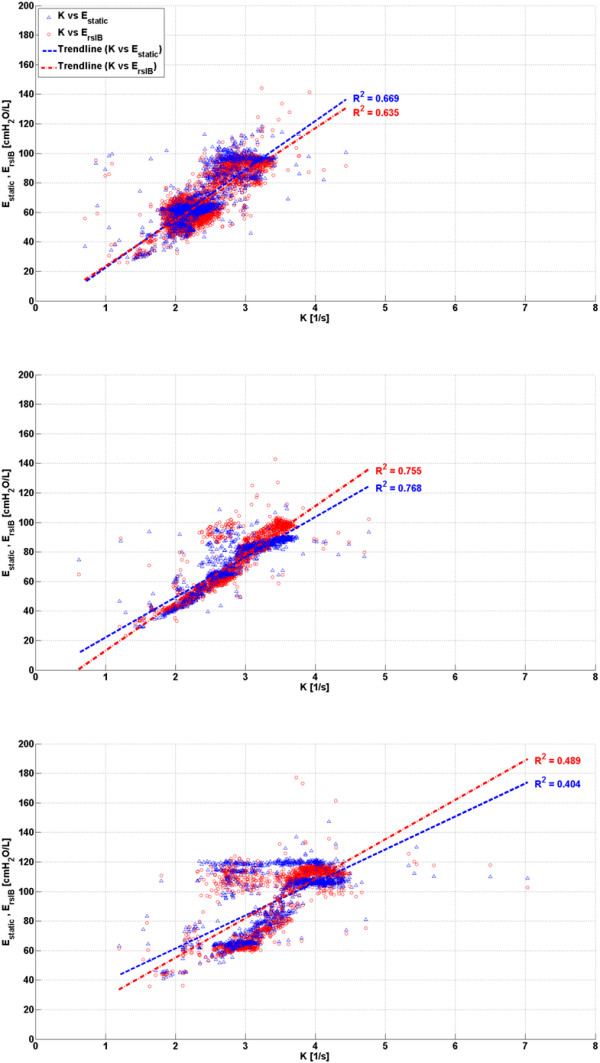
**Correlation between *K *and validation metrics *E***_***static***_**and *E***_***rsIB***_**.** Subject 1 (Top), Subject 2 (Middle) and Subject 3 (Bottom).

Performance was assessed by trend correlation coefficient (R^2^) across all three phases where comparison between *K* and *E*_*static*_, and *K* and *E*_*rsIB*_ values were made for each breathing cycle. From Figure 7, it can be seen that Subject 3 has the lowest correlation coefficients across both validation metrics, while Subject 2 has the highest correlation coefficients as expected from the observation in Figures 4, 5 and 6. In this study, Subject 3 has the largest body mass and reached the highest severity of ARDS while Subject 2 has the lowest body mass and reached lowest severity of ARDS. Thus, it is possible that increased body mass and/or severity of ARDS may influence the physiological process of expiration, leading to a lower observed correlation between the system time-constant, *K*, and the elastance of the lung. However, due to the limited number of trials in this study, this conclusion is limited in its impact and warrants further investigation.

### Outcomes

The time-constant model has demonstrated its performance in continuous monitoring of respiratory mechanics as disease state progresses in fully sedated experimental piglets. The overall median fitting error was comparable to that obtained using the EIP method. However, variable respiratory resistance may lead to a lower correlation between the system time-constant, *K*, and the elastance of the lung. The resistance was found to vary in Subject 3 during phase 2 and Subjects 2 and 3 during phase 3, based on the results of the integral-based method. Thus, a larger study cohort is required to further validate this method. However, it should be noted that if resistance is varying, it can be identified and accounted for. In general, the time-constant model was able to provide clinically relevant physiological insight not readily available at the bedside to guide MV therapy.

One potential application of the time-constant model is to estimate respiratory mechanics of spontaneously breathing patients which have individual breathing efforts aside from ventilator support [36], significantly altering the respiratory mechanics. In this case, oesophageal pressure measurements are required to determine patient-specific respiratory mechanics during inspiration [16]. However, this technique is considered uncomfortable for the patient and its application is limited in daily monitoring despite its potential to guide MV for ARDS patients [37,38]. Expiration is hypothesised to be primarily or completely passive, regardless of whether the patient is sedated or spontaneously breathing. Thus, muscle activity is assumed to be absent or relatively minimal [11,36]. Therefore, it may be possible to determine real-time lung parameters for spontaneously breathing patients without additional measuring tools, opening up the clinical applicability of a model-based approach to guiding MV therapy. However, application of the time constant model in tracking respiratory mechanics in spontaneously breathing patients warrants further investigation.

### Limitations

Pathogenesis of ARDS animal models are more consistent where the methods of developing ARDS is known and controlled. In contrast, ICU patients are more variable as the causes of disease are different with greater inter-patient variability in response to treatment. Thus, the application of the time-constant model to human patients warrants further investigation.

The EIP method may be erroneous when the automated EIP is too short and does not allow peak pressure to drop to the true plateau pressure [39]. In addition, this simple two-point, static approach may be too simplistic to capture some finer aspects of lung mechanics. Hence, no elastance metric is necessarily a gold standard. This analysis is predominantly based on the comparison of trends where each subject is their own reference. Thus, the best validation of a model is the ability to track clinically expected trends.

The estimation of respiratory resistance and the effect of ARDS on this parameter may be limited. Airway collapse alters respiratory resistance [12,14,19,30]. However, this change is less significant compared to changes in respiratory elastance due to alveolar collapse. A collapsed airway will not have air entering and thus, resistance on expiration will not exist. Equally, a nearly closed airway will have higher resistance. Both hypotheses are potential effects from ARDS, but result in contradiction.

The clinical merit of *K* relies on the assumption that the respiratory resistance remains constant throughout treatment. Significant variation will result in a poor correlation between *K* and respiratory elastance. However, the degree to which respiratory resistance varies with PEEP and disease state was different for each of the three subjects in this study. A larger cohort may provide more consistency.

The time-constant model used in this study may not accurately capture regional differences in mechanical properties [16]. Passive expiration could be more accurately modelled using a bi-exponential function, combining the effects of a slower and a faster time-constant to effectively model each lung separately [16,40,41]. However, each time-constant cannot be uniquely distinguished. Therefore, this method could not track disease progression within each lung separately.

## Conclusions

Expiratory data is normally neglected by conventional metrics of respiratory system mechanics. However, the time-constant model provides an alternative means to track changes in disease state throughout treatment to optimise MV. Setting PEEP at minimum elastance, i.e. minimum time-constant, theoretically provides optimal PEEP. In general, the trends obtained using the time-constant model matched those obtained using the EIP method and the integral-based method, demonstrating robustness and potential for guiding MV therapy. However, the assumption of constant resistance leads to less physiological insight.

These are the first results to track and identify clinically relevant and expected pulmonary mechanics breath-to-breath through a clinical recruitment manoeuvre. Such tracking offers insight beyond the metrics and methods presented. Overall, further research is required to confirm the use of such real-time methods in actual ARDS patients, both sedated and spontaneously breathing. However, the ability to identify and track clinically relevant responses to disease progression and MV in real-time shows significant new potential.

## Abbreviations

ARDS: Acute respiratory distress syndrome; ICU: Intensive care unit; MV: Mechanical ventilation; PEEP: Positive end expiratory pressure; EIP: End-inspiratory pause; PIP: Peak inspiratory pressure; IQR: Inter-quartile range.

## Competing interests

The authors declare that they have no competing interests.

## Authors’ contributions

EJD assisted in the development of the time-constant model, performed the validation and drafted the manuscript. YSC participated in the implementation of the clinical trials, assisted in the development of the time-constant model and helped to draft the manuscript. JGC participated in the implementation and coordination of the study and helped to draft the manuscript. GMS participated in the implementation and coordination of the study. BL and NJ implemented the clinical trials. NSD assisted in the development of the time-constant method. TD participated in the implementation of the clinical trials and the implementation and coordination of the study. All authors read and approved the final manuscript.
